# Enhancement of Glucose Uptake in Mouse Skeletal Muscle Cells and Adipocytes by P2Y_6_ Receptor Agonists

**DOI:** 10.1371/journal.pone.0116203

**Published:** 2014-12-30

**Authors:** Ramachandran Balasubramanian, Bernard Robaye, Jean-Marie Boeynaems, Kenneth A. Jacobson

**Affiliations:** 1 Molecular Recognition Section, Laboratory of Bioorganic Chemistry, National Institute of Diabetes and Digestive and Kidney Diseases, National Institutes of Health, Bethesda, Maryland, United States of America; 2 Institute of Interdisciplinary Research, IRIBHM, Université Libre de Bruxelles, Gosselies, Belgium; 3 Université Libre de Bruxelles, Brussels, Belgium; USDA-ARS, United States of America

## Abstract

Glucose uptake by peripheral tissues such as skeletal muscles and adipocytes is important in the maintenance of glucose homeostasis. We previously demonstrated that P2Y_6_ receptor (P2Y_6_R) agonists protect pancreatic islet cells from apoptosis and stimulate glucose-dependent insulin release. Here, we investigated the effects of P2Y_6_R activation on glucose uptake in insulin target tissues. An agonist of the P2Y_6_R, P^1^-(5′-uridine)-P^3^-(5′-*N^4^*-methoxycytidine)-triphosphate (MRS2957), significantly increased the uptake of [^3^H]2-deoxyglucose in mouse C2C12 myotubes and 3T3-L1 adipocytes, and this stimulation was significantly decreased by a selective P2Y_6_R antagonist *N*,*N*″-1,4-butanediyl-*bis*[*N′*-(3-isothiocyanatophenyl)thiourea] (MRS2578). Pre-incubation with Compound C (an inhibitor of 5′-AMP-activated protein kinase, AMPK), or AMPK siRNA abolished the stimulatory effect of MRS2957 on glucose uptake. Also, MRS2957 (60 min incubation) increased recruitment of the facilitated glucose transporter-4 (GLUT4) to the cell membrane, which was blocked by MRS2578. Treatment of C2C12 myotubes with MRS2957 induced significant phosphorylation of AMPK, which increase GLUT4 expression through histone deacetylase (HDAC)5 signaling. Glucose uptake in primary mouse adipocytes from wild-type mice was stimulated upon P2Y_6_R activation by either MRS2957 or native agonist UDP, and the P2Y_6_R effect was antagonized by MRS2578. However, in adipocytes from P2Y_6_R-knockout mice P2Y_6_R agonists had no effect on glucose uptake, and there was no change in the glucose uptake by insulin. Our results indicate that the P2Y_6_R promotes glucose metabolism in peripheral tissues, which may be mediated through AMPK signaling.

## Introduction

Type 2 diabetes (T2D) has become a common and serious global health problem due to genetic factors and changes in diet and life style. Insulin-stimulated glucose uptake is severely impaired in T2D, which may be due to insulin resistance in skeletal muscle and adipocytes, a key feature of T2D. Along with this insulin resistance, a relevant role in maintaining hyperglycemia is played by an impaired basal glucose uptake, which was reduced by>30% in relatively non-insulin sensitive organs such as kidney and skin [1]. Hence the identification of novel pathways that can increase glucose uptake independent of insulin signaling pathways is of great therapeutic interest.

**Figure 1:**
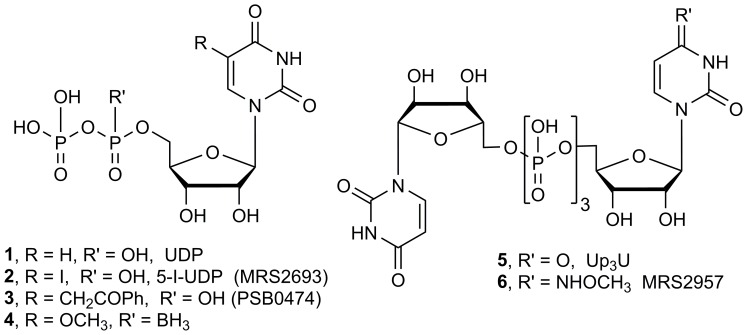
Structures of P2Y_6_R ligands: nucleotide agonists.

Extracellular nucleotides act as signaling molecules in many physiological processes by activating cell surface P2 receptors [2]. The P2Y family of G protein-coupled receptors, which are activated by various endogenous mono- and dinucleotides, has a role in processes related to diabetes. The P2Y_6_ receptor (P2Y_6_R) is a G_q_-coupled receptor that is activated by uridine 5′-diphosphate (UDP **1**, Fig. 1) and acts through phospholipase Cβ to induce inositol lipid signaling. It is highly expressed in dendritic cells, cardiomyoctes, liver, placenta and other sites [3]. Also, P2Y_6_R mRNA is expressed in high levels in smooth muscle cells, kidney and spleen, and in moderate levels in lung, intestine, adipose tissue, bone and heart [4]. P2Y_6_R activation is associated with antiapoptotic effects dependent on protein kinase C and extracellular signal-regulated kinases in skeletal muscle and pancreatic β-islet cells [5]–[7]. The cytoprotection induced by UDP acting at the P2Y_6_R is not observed with other G_q_-coupled P2YRs, e.g. the action of UTP at the P2Y_4_R or 2-methylthio-ADP at the P2Y_1_R. Furthermore, UDP and other P2Y_6_R agonists increase glucose-dependent release of insulin from mouse pancreatic β-islet cells (MIN6) and primary mouse islets [7], [8]. Endogenous UDP has been shown to activate P2Y_6_R to contribute to insulin secretion in vivo in the mouse [9]. A role for the ADP-responsive P2Y_13_R in pancreatic β-islet cells has also been demonstrated [10].

The structure activity relationships (SARs) at the P2Y_6_R have been explored through the synthesis of nucleotide agonists and non-nucleotide antagonists [11]–[14]. Various pyrimidine nucleotides have been reported as selective agonists, e.g. nucleoside 5′-diphosphates **2**–**4** and dinucleoside (5’,5’)-triphosphates **5** and **6** [12]–[14], while the only selective antagonists yet reported are irreversibly acting isothiocyanate derivatives [11]. In our recent work, we have reported that P2Y_6_R-selective agonist P^1^-(5′-uridine)-P^3^-(5′-*N^4^*-methoxycytidine)-triphosphate (MRS2957, **6**) activates 5′-AMP-activated protein kinase (AMPK) in MIN6 mouse β-islet cells through a calmodulin-dependent protein kinase kinase (CaMKK)β signaling pathway [7]. AMPK has been widely reported as a target for treatment of T2D, and it acts indirectly to increase insulin-independent glucose uptake [15]. Activation of AMPK by 5-amino-1-β-D-ribofuranosyl-imidazole-4-carboxamide (AICAR) increases glucose uptake in skeletal muscle in the diabetic mouse and human, independent of insulin signaling [16], [17]. Apart from regulation of cellular energy pathways, AMPK also increases translocation of the facilitated glucose transporter-4 (GLUT4) and thus promotes glucose uptake in skeletal muscle [18].

Other G_q_-coupled receptors are known to promote glucose uptake in insulin target tissues. For example, glucose uptake was stimulated by activation of the endothelin-1 receptor in 3T3-L1 adipocytes and the M_3_-muscarinic acetylcholine receptor in L6 skeletal muscle cells, an effect dependent on both CaMKK and AMPK [15], [19]. The effects of purinergic agonists on glucose uptake have been explored, but the association with specific receptor subtypes, either P2YRs or P2X ion channels has not yet been established [20]. In this context, we characterized the effects of P2Y_6_R activation on glucose uptake in skeletal muscle and adipocytes and the involvement of AMPK and other associated signaling pathways.

## Results

We selected two cell lines for examination of the effects of P2Y_6_R agonists on glucose uptake in target tissues: C2C12 mouse skeletal myoblasts and 3T3-L1 mouse fibroblasts that differentiate into adipocytes. We already demonstrated a pharmacological effect of the P2Y_6_R in C2C12 cells, i.e. protection against TNFα-induced apoptosis [6].

### P2Y_6_R agonist stimulates glucose uptake

**Figure 2:**
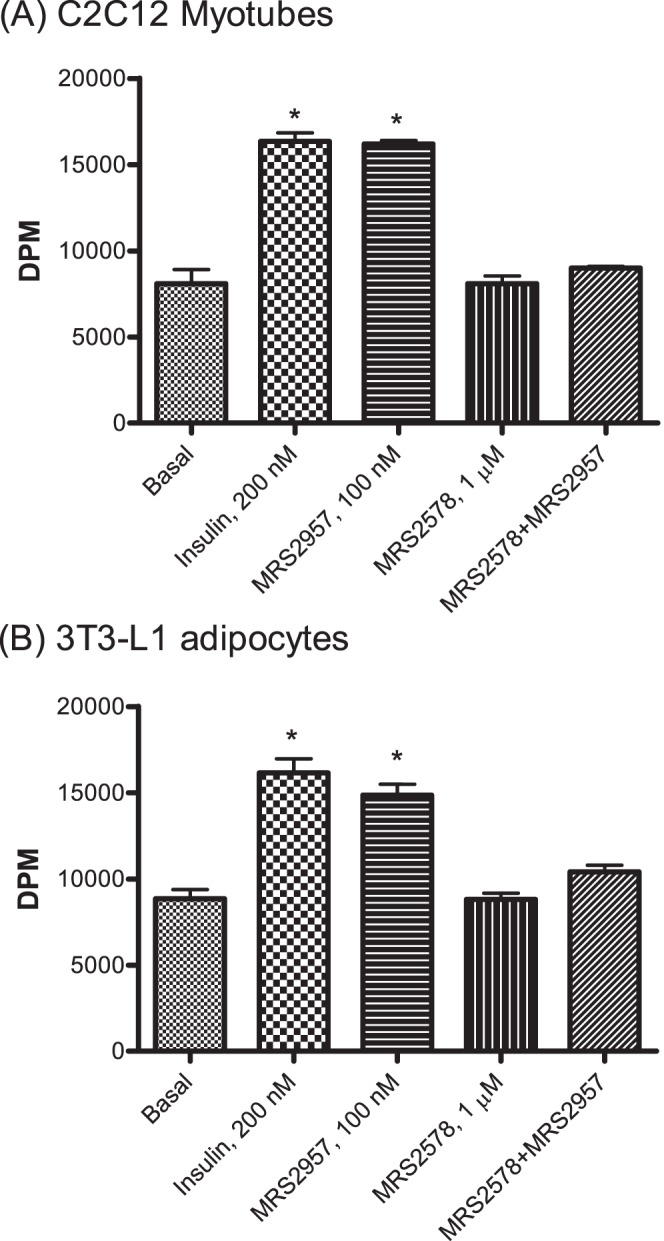
(A and B). Effects of P2Y_6_R agonist MRS2957 on glucose uptake in C2C12 cells (A) and 3T3-L1 cells (B). MRS2957 was applied in the presence or absence of P2Y_6_R antagonist MRS2578 (1 µM). *P<0.05, when compared to controls (n = 3).

Glucose is the key energy source in skeletal muscle, and glucose transport is the rate-determining step in glucose-dependent intracellular energy production. We evaluated the efficacy of P2Y_6_R agonist MRS2957 to stimulate glucose uptake in differentiated C2C12 cells (Fig. 2A) and in 3T3-L1 adipocytes (Fig. 2B). Treatment with MRS2957 (100 nM) significantly increased glucose uptake in both C2C12 cells and 3T3-L1 adipocytes when compared to control cells. In 3T3-L1 adipocytes, a concentration-dependent increase was observed upon treatment with 100 nM MRS2957. Also, glucose uptake induced by MRS2957 (100 nM) was not significantly different from that induced by insulin (200 nM). The effect of the P2Y_6_R agonist was blocked by pre-treatment with P2Y_6_R antagonist *N*,*N*″-1,4-butanediyl-*bis*[*N′*-(3-isothiocyanatophenyl)thiourea] (MRS2578, 1 µM) in both cell lines. This shows that the increase in glucose uptake by MRS2957 occurs through activation of the P2Y_6_R. The concentration of MRS2957 at 100 nM was chosen based on our studies with various concentrations of MRS2957 (S1 Fig.). Glucose uptake in cells co-treated with insulin and MRS2957 did not show any significant difference when compared to cells treated with insulin alone (S2 Fig.).

### Role of AMPK in P2Y_6_R-mediated glucose uptake

**Figure 3:**
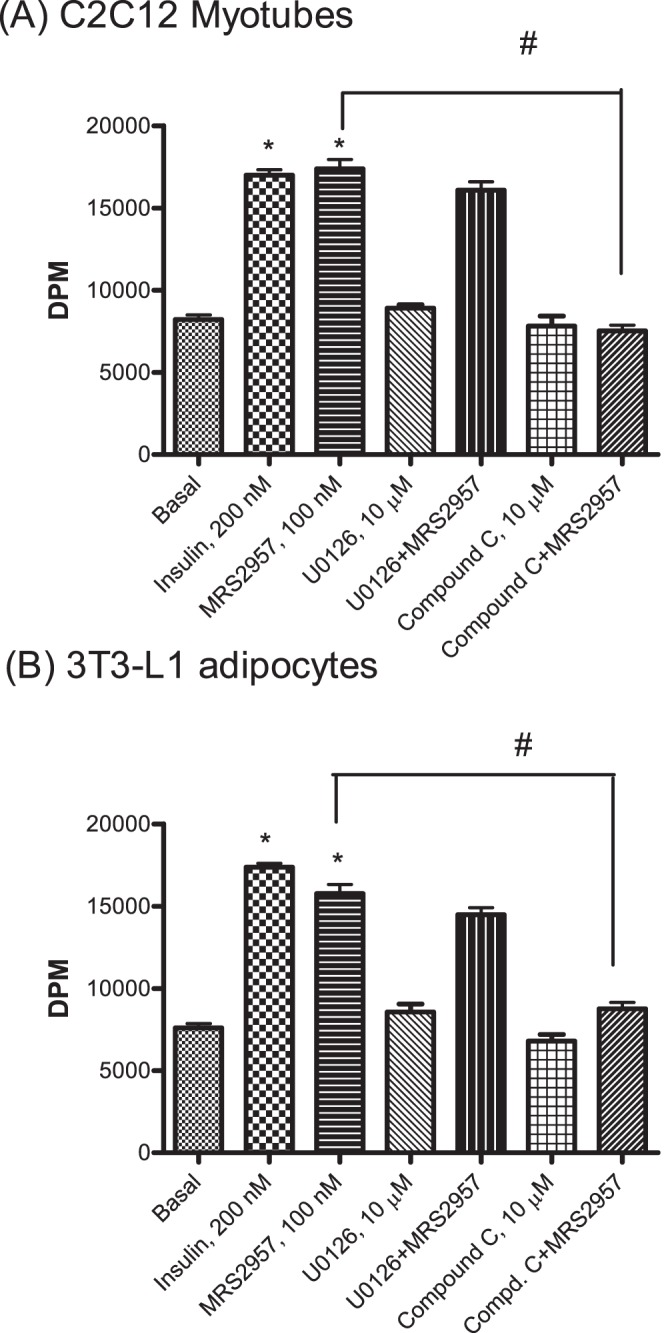
(A and B). Effects of inhibitors of intracellular signaling pathways on P2Y_6_R-mediated glucose uptake. C2C12 cells (A) or 3T3-L1 cells (B) were preincubated with inhibitors of MAPK (U0126, 10 µM) and AMPK (Compound C, 10 µM) followed by treatment with P2Y_6_R agonist MRS2957. * *P*<0.05, when compared to controls, ^#^ *P*<0.05, when compared to MRS2957, 100 nM (n = 3).

**Figure 4:**
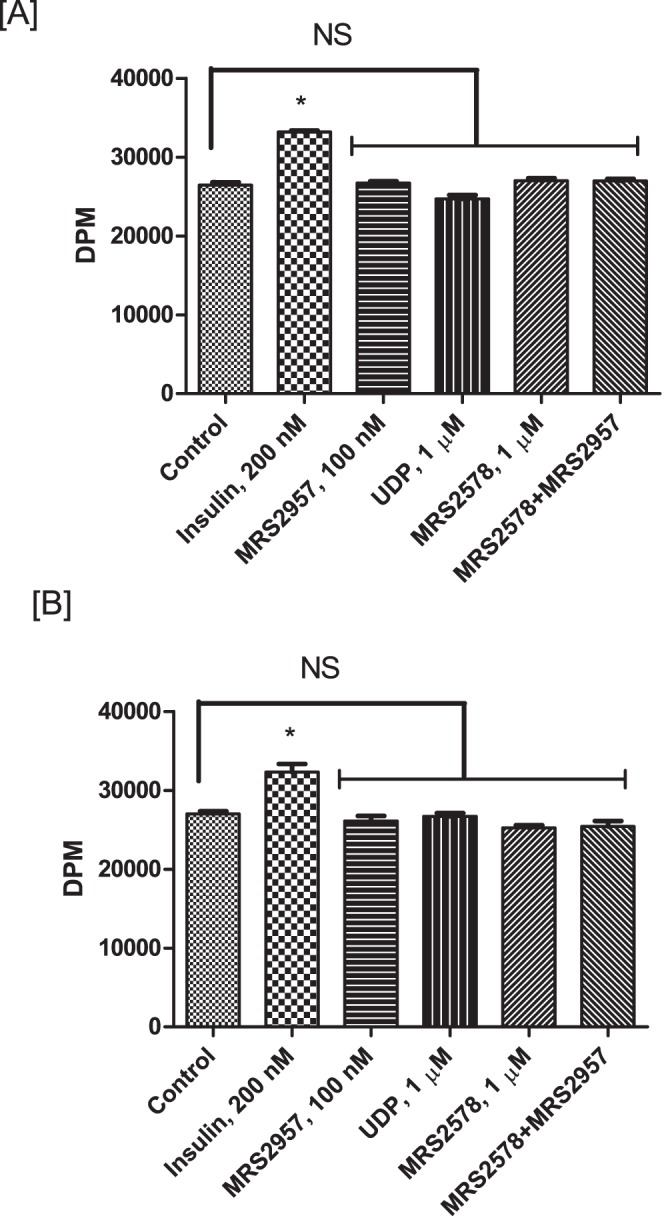
Glucose uptake in C2C12 myotubes (A) and 3T3-L1 adipocytes (B) transfected with AMPK siRNA. *P<0.05, when compared to controls, NS, non-significant when compared to controls (n = 3).

To explore the mechanism involved in the P2Y_6_R-mediated glucose uptake, we evaluated AMPK and mitogen-activated protein kinases (MAPK) in myotubes and adipocytes. P2Y_6_R ligands have already been reported to activate MAPK [6] and AMPK [7], which are known to be involved in glucose uptake [17]. We used inhibitors of MAPK (1,4-diamino-2,3-dicyano-1,4-bis-(2-aminophenylthio)butadiene, U0126, 10 µM) and AMPK (6-[4-(2-piperidin-1-yl-ethoxy)-phenyl)]-3-pyridin-4-yl-pyrrazolo[1,5-a]-pyrimidine, Compound C, 10 µM) to study their effect on P2Y_6_R-mediated glucose uptake. Preincubation with MAPK inhibitor U0126 did not affect the P2Y_6_R agonist-induced glucose uptake in either C2C12 myotubes (Fig. 3A) or 3T3-L1 adipocytes (using MRS2957, Fig. 3B). In contrast, preincubation with Compound C followed by treatment with MRS2957 significantly reduced glucose uptake when compared to cells treated with MRS2957 alone. The effects of U0126 or Compound C alone did not differ from control. This shows that AMPK is involved in glucose uptake induced by the P2Y_6_R agonist MRS2957. To further confirm this result we also used small interfering RNA (siRNA) for AMPK to silence the expression of AMPK in C2C12 myotubes and 3T3-L1 adipocytes (Fig. 4A and 4B). In the absence of AMPK, treatment with MRS2957 failed to increase glucose uptake in both of the cell lines. Thus, we were able to confirm the role of AMPK in P2Y_6_R-mediated glucose uptake. Also, there were no changes in MRS2957-induced glucose uptake in both the cell lines when transfected with a scrambled siRNA control (S3 Fig.).

**Figure 5:**
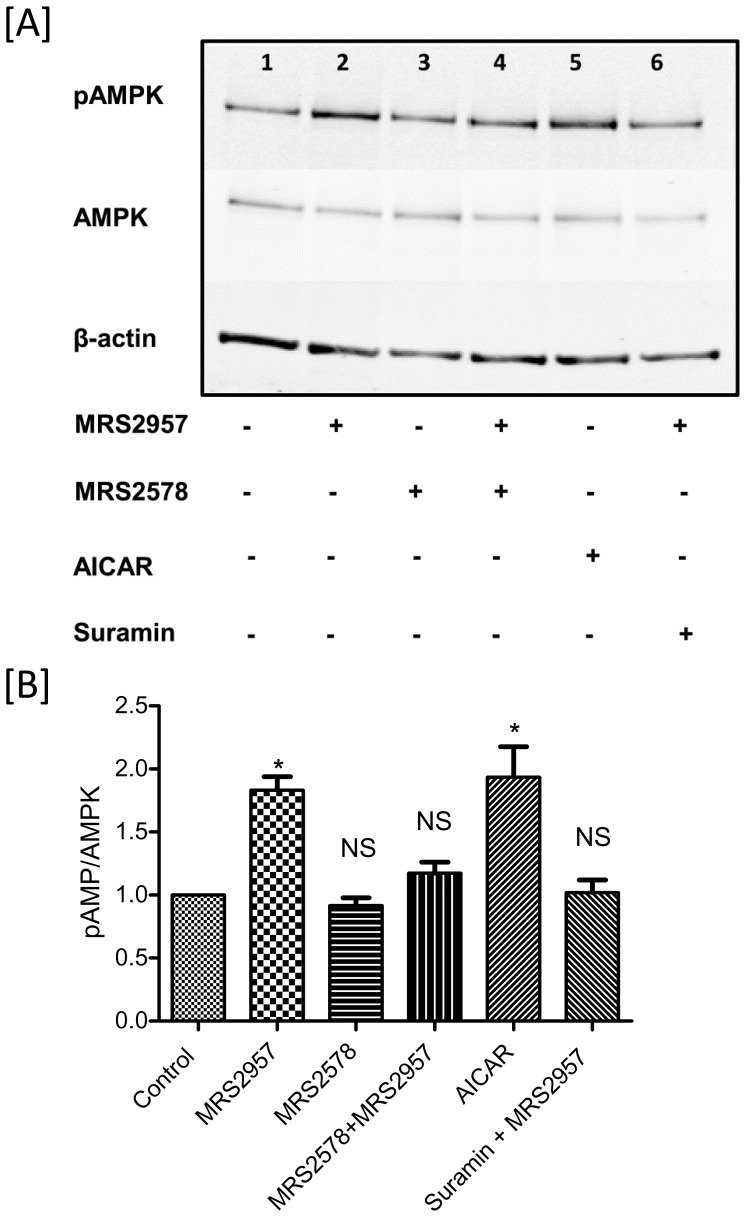
Phosphorylation of AMPK in C2C12 myotubes (A and B) after treatment with MRS2957. This is a representative blot from three individual experiments. The density of the bands were quantified and compared to the controls (B and D). *P<0.05, when compared to controls, NS, non-significant when compared to controls (n = 3). **Lane 1**, Control, **2**, MRS2957 (100 nM), **3**, MRS2578(1 µM), **4**, MRS2578+ MRS2957, **5**, AICAR (200 µM), **6**, Suramin (20 µM)+MRS2957.

**Figure 6:**
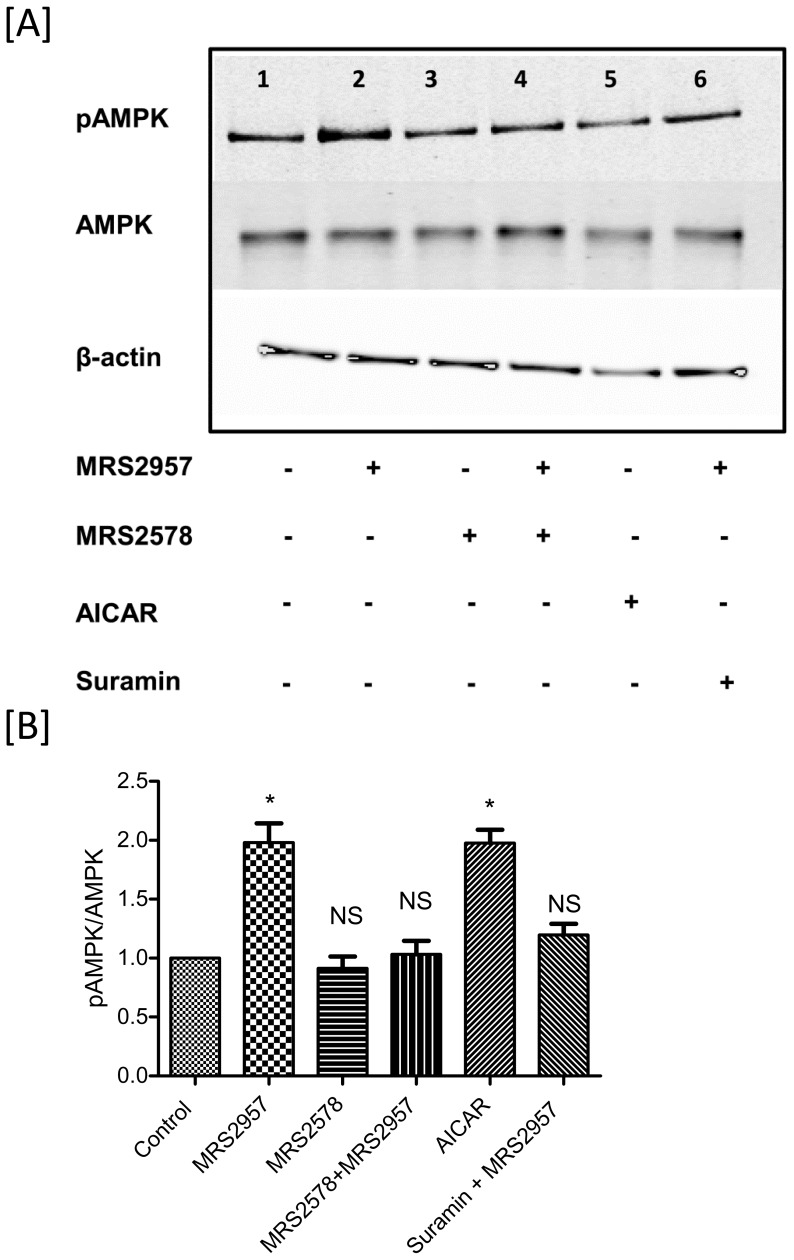
Phosphorylation of AMPK in 3T3-L1 adipocytes (A and B) after treatment with MRS2957. This is a representative blot from three individual experiments. The density of the bands were quantified and compared to the controls (B and D). *P<0.05, when compared to controls, NS, non-significant when compared to controls (n = 3). **Lane 1**, Control, **2**, MRS2957 (100 nM), **3**, MRS2578(1 µM), **4**, MRS2578+ MRS2957, **5**, AICAR (200 µM), **6**, Suramin (20 µM)+MRS2957.

We have previously demonstrated the efficacy of MRS2957 in the activation of AMPK in MIN6 cells [7]. Now, we examined the ability of MRS2957 to activate AMPK in both C2C12 myotubes (Fig. 5) and 3T3-L1 adipocytes (Fig. 6). Differentiated cells were treated with MRS2957 (100 nM) with or without MRS2578 (1 µM) for 1 h, and the cells were lysed and proteins were immunoblotted and probed with phospho-AMPK antibody. Treatment with MRS2957 resulted in significant phosphorylation of AMPK in both of the cells lines. This shows that AMPK signaling is involved in P2Y_6_R-mediated glucose uptake in skeletal muscle and differentiated adipocyte cell lines.

### GLUT4 Translocation

**Figure 7:**
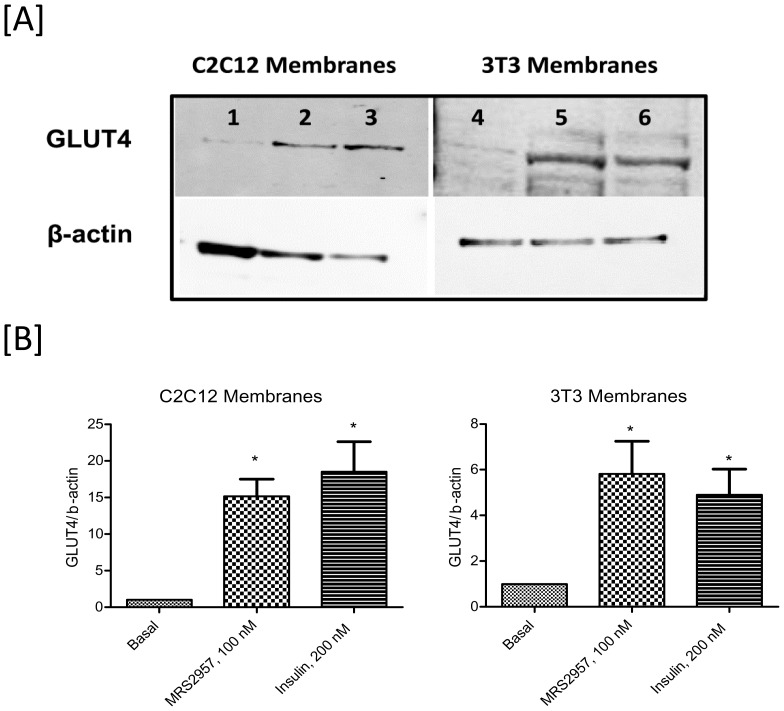
Translocation of glucose transporters GLUT4 in the plasma membrane upon activation of P2Y_6_R. (A) Immunoblotting is used to determine the changes in the membrane levels of the GLUT4 transporter. This is a representative blot from three individual experiments. Key to lanes: **1**–**3**, C2C12 myotubes; **4**–**6**, 3T3-L1 adipocytes. **1** and **4**, controls; **2** and **5**, MRS2957, 100 nM; **3** and **6**, 200 nM insulin. (B) GLUT4/β-actin ratio based on the quantitation of the bands.

In the presence of appropriate extracellular stimuli, GLUT1 and GLUT4 are recruited to the plasma membrane and/or activated to facilitate glucose transport [21]. To evaluate if P2Y_6_R recruits glucose transporters in C2C12 cells and 3T3-L1 adipocytes, we examined by Western blotting the changes in the levels of glucose transporter GLUT4 in the plasma membrane upon activation of P2Y_6_R. As shown in Fig. 7 (A&B), the plasma membrane localization of GLUT4 is high in adipocytes treated with MRS2957 when compared to that of the control adipocytes.

### Glucose uptake in primary adipocytes by P2Y_6_R activation

**Figure 8:**
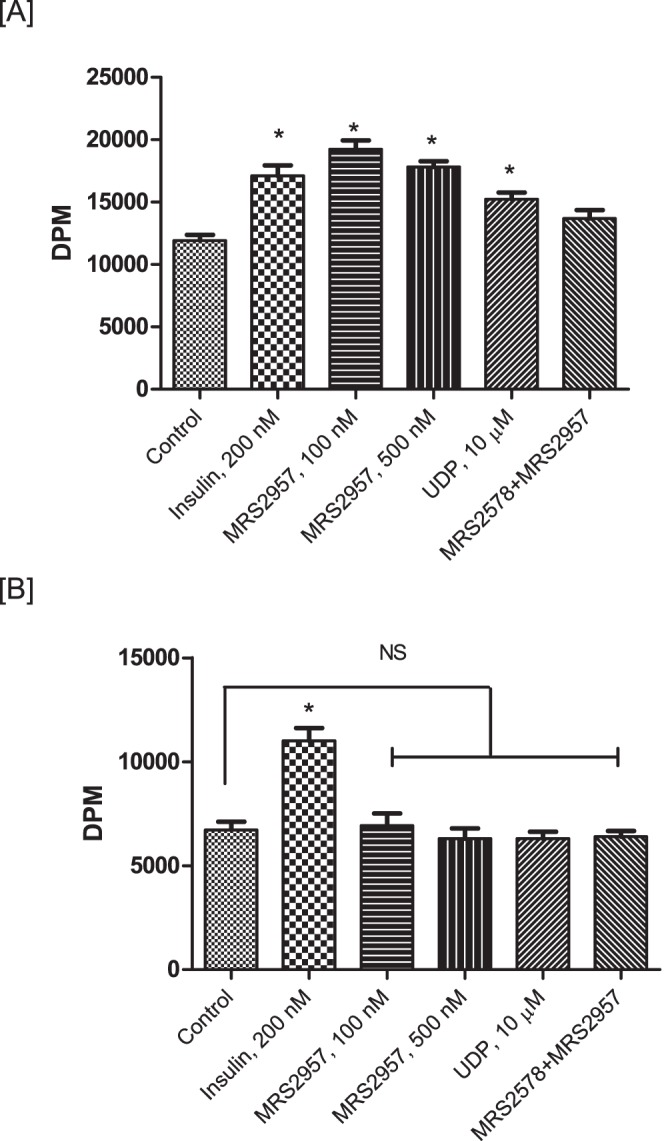
Glucose uptake assay in primary adipocytes isolated from WT (A) and P2Y_6_R-KO mice (B). *P<0.05, when compared to controls, NS, non-significant when compared to controls (n = 3).

To evaluate the efficacy of a P2Y_6_R agonist in increasing glucose uptake in primary mouse adipocytes we isolated adipocytes from both wild-type (WT) and P2Y_6_R-knockout mice. The stromal vascular fraction (SVF) of adipose tissue, a rich source of preadipocytes, was isolated from mouse using the procedure described in the Materials and Methods section. The preadipocytes were differentiated into adipocytes and used to evaluate the efficiency of P2Y_6_R agonists UDP and MRS2957 to stimulate glucose uptake. In adipocytes from WT mice (Fig. 8A), MRS2957 treatment resulted in a significant increase in glucose uptake when compared to basal levels. Moreover, preincubation with MRS2578 abated the increase in glucose uptake by MRS2957. In the case of adipocytes isolated from P2Y_6_-knockout (KO) mice (Fig. 8B), there was no significant difference in glucose uptake between MRS2957-treated and control adipocytes. Note, however, that insulin-stimulated glucose uptake was intact in the adipocytes from KO mice. This demonstrates that P2Y_6_R activation may lead to increased glucose uptake in primary adipocytes. Also, we observed a decrease in the basal glucose uptake in P2Y_6_R KO primary adipocytes when compared to that of the WT, although the significance of this difference is not clear.

### Effects of AMPK on HDAC5

**Figure 9:**
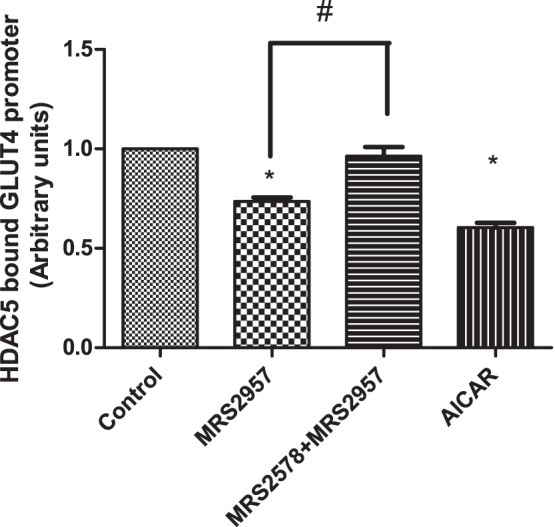
HDAC5 associated GLUT4 promoter DNA in the MEF2 binding region following treatment with MRS2957 (100 nM) or AICAR (200 µM) or MRS2578 (1 µM)+MRS2957 in C2C12 myotubes using ChIP assay. *P<0.05, when compared to controls, ^#^ *P*<0.05, when compared to MRS2578+ MRS2957 (n = 3).

AMPK is known to stimulate GLUT4 transcription by phosphorylating the enzyme histone deacetylase 5 (HDAC5), thereby preventing its association with the GLUT4 promoter and the resulting repression of transcription [22]. Our results show that the P2Y_6_R activates AMPK, which results in increased glucose uptake. To determine if the mechanism involves HDAC5 regulation of the GLUT4 pathway, we performed a chromatin immunoprecipitation (ChIP) assay using C2C12 myotubes treated with MRS2957, MRS2578+MRS2957 or AICAR, and the chromatin was immunoprecipitated using an anti-HDAC5 antibody. The DNA was amplified using primers for HDAC5 associated with the GLUT4 promoter surrounding the region for the myocyte enhancer factor (MEF) 2. The gene amplification by qPCR indicated that the association of HDAC5 with the GLUT4 promoter was significantly reduced in MRS2957-treated C2C12 cells, and similar results were observed in C2C12 myotubes treated with AMPK activator AICAR (Fig. 9). Also, preincubation with antagonist MRS2578 followed by treatment with MRS2957 reversed the decrease in GLUT4 promoter association to HDAC5. These results are consistent with our hypothesis that activation of P2Y_6_R activates AMPK, which inhibits HDAC5 and consequently results in increased GLUT4 expression in C2C12 myotubes.

## Discussion

We demonstrate here that glucose uptake in skeletal myocytes and adipocytes in culture is increased in the presence of a P2Y_6_R agonist MRS2957. This agonist is more potent than UDP at the human P2Y_6_R (EC_50_ 12 nM, compared to 300 nM for UDP) [12]. Glucose uptake in primary mouse adipocytes was also stimulated upon P2Y_6_R activation by UDP and MRS2957. Thus, a P2Y_6_R agonist would be expected to produce beneficial effects for diabetes both with respect to insulin release from the pancreas and glucose homeostasis in certain insulin target tissues. However, it is not clear if this approach is clinically relevant, because proinflammatory effects of P2Y_6_R activation, such as in the allergic airway and the vasculature, have also been reported [23]–[26].

A role for the regulation of GLUT4 expression by HDAC in both skeletal muscle cells and mature adipocytes has been reported previously [22], [27]–[30]. A conserved binding site for the MEF2 transcription factor is essential for the normal expression of skeletal muscle GLUT4. A direct correlation between reduced MEF2 expression and reduced GLUT4 expression has been reported. Studies have also shown a link between class II histone deacetylases (HDACs) in the regulation of GLUT4 expression in various cell types. HDAC5 acts as a repressor of GLUT4 expression by its association with MEF2. It has also been reported that AMPK phosphorylates HDAC5 to convert it to its inactive form, and consequently to increase GLUT4 expression [22]. Our study using a ChIP assay showed that treatment with MRS2957 decreased HDAC5 occupancy of the GLUT4 promoter region in C2C12 myotubes. Based on this result, we hypothesized that treatment with MRS2957 activates AMPK, which inactivates HDAC5 by phosphorylation and thus results in increased GLUT4 expression leading to increased glucose uptake. HDAC inhibition has been suggested for the treatment of diabetes [31], further emphasizing the possible disease relevance of the inhibitory influence of P2Y_6_R activation.

The action of UDP at the P2Y_6_R was not previously implicated in glucose uptake in insulin target tissues, but nucleotide effects through other P2 receptors were reported. Previous reports initially indicated that activation of the G_q_-coupled P2Y_2_R might be beneficial in diabetes by stimulating glucose uptake [32], [33]. Extracellular ATP enhanced glucose transport in human fibroblasts via GLUT1, but the P2Y-dependent activation of GLUT1 was found to be deficient in fibroblasts from T2D individuals [33]. Also, the vasodilatory response to P2Y_2_R activation by ATP or UTP is severely attenuated in diabetic patients [34]. Thus, a P2Y_2_R agonist is not likely to be useful for treatment of diabetes because of changes in the receptor responsiveness in the pathological state. Also, side effects of these nucleotides might interfere with their beneficial effects. For example, ATP enhanced uptake by Na^+^-glucose cotransporters in rabbit renal proximal tubule cells in a manner dependent on P2YRs, G_i_ protein, 3′-5′-cyclic adenosine monophosphate and p38 MAPK [35]. Thus, this P2Y_2_R-dependent activation of Na^+^-glucose cotransporters would be detrimental in diabetes. Recently, it was suggested that activation of other classes of G_q_-coupled receptors present in the β-islet cells and target tissues might be similarly advantageous [36], [37]. The stimulation of a G_q_-coupled M_3_ muscarinic acetylcholine receptor was shown to be beneficial with respect to pancreatic β-cells and liver in diabetic animal models. It is not known if the G_q_-coupled P2Y_6_R responses are maintained in diabetic patients or whether the proinflammatory responses, vasoconstriction and other detrimental effect already ascribed to P2Y_6_R activation [23]–[25] would preclude use of such agonists in diabetes treatment.

Nevertheless, we have found that P2Y_6_R activation by both UDP and a synthetic potent and selective agonist enhanced glucose transport in two different cell lines and primary cells from mouse. This suggests a potential benefit with respect to defective glucose metabolism, which is known to be associated with diabetes in peripheral tissues such as skeletal muscles and adipocytes. The effect of P2Y_6_R agonists on glucose uptake in other target tissues, such as the brain and liver, remain to be determined. This phenomenon needs to be explored in vivo and particularly in diabetic models in order to establish its relevance to diabetes treatment.

## Materials and Methods

### Materials

P2Y_6_R antagonist MRS2578 and P2Y_6_R agonist MRS2957 were purchased from Tocris Bioscience (Ellisville, MO) or prepared as described [12]. Cytochalasin-B, Compound C (dorsomorphin) and U0126 monoethanolate were from Sigma-Aldrich (St. Louis, MO). Antibodies to phospho-AMPK, AMPKα1/2, and anti-mouse and anti-rabbit horseradish peroxidase-conjugated antibodies were purchased from Cell Signaling Technologies (Danvers, MA). P2Y_6_R specific antibody was from Alomone Labs Ltd. (Jerusalem, Israel). Goat anti-mouse IRDye 680LT and Goat anti-rabbit IRDye 800 CW were purchased from Li-Cor BioSciences (Lincoln, NE). All other reagents not mentioned were of the highest grade commercially available.

### Cell culture

C2C12 skeletal muscle cells (American Type Culture Collection, Manassas, VA) were grown at 37°C in Dulbecco’s modified Eagle’s medium (DMEM) containing 10% fetal bovine serum until they reached 60–70% confluence and after which they were differentiated to myotubes using 2% horse serum and used for the experiments. 3T3-L1 mouse fibroblasts (American Type Culture Collection, Manassas, VA) were grown in DMEM containing 10% fetal bovine serum until they reached 70% confluence. Later, 3T3-L1 cells were differentiated into adipocytes using 0.5 mM 3-isobutyl-1-methylxanthine, 1 µM dexamethasone, 5 µg/ml insulin and 1 µg/ml thiazolidinedione for two days followed by incubation for 3 days with DMEM containing insulin (5 µg/ml).

### [^3^H]2-Deoxy-D-glucose uptake assay

Uptake of [^3^H]2-deoxyglucose (PerkinElmer, Boston, MA) was measured in 3T3-L1 adipocytes and C2C12 myocytes differentiated in 24-well plates. After culture, cells were washed twice with serum-free DMEM and incubated in the serum-free DMEM for 2 h at 37°C. Cells were then washed twice in PBS and then incubated in Krebs ringer bicarbonate buffer containing 0.3% BSA in the presence or absence insulin or agonists and/or antagonists for 30 min at 37°C. Antagonists and inhibitors were added 30 min prior to the addition of P2Y_6_R agonist. After agonist treatment, uptake of 10 µM [^3^H]2-deoxyglucose was measured over 5 min. Reactions were terminated by rapidly washing the cells twice with ice-cold Krebs ringer bicarbonate buffer. Cell were then extracted by using 0.2% SDS, and the aliquots from the cell extracts were counted by liquid scintillation and used to determine protein concentration. Nonspecific uptake was measured in the presence of 10 µM of cytochalasin-B, and the values were subtracted from those of the specific binding.

### Gene silencing by siRNA transfection

AMPK siRNA (Santa Cruz Biotechnology, Dallas, TX) were used to silence the expression of AMPK gene in both C2C12 and 3T3-L1 cells by employing reverse transfection method. The complex of siRNA and transfection reagent was mixed with either fully confluent C2C12 cells or differentiated 3T3-L1 adipocytes and then transfected cells were allowed to grow for 2–3 days. The transfection was confirmed by Western blot against AMPK antibody. The transfected cells were used for glucose uptake assay. A control siRNA transfected cells were used as a control for the experiment.

### Isolation of adipocytes from mouse

Animal experiments were approved by NIDDK, National Institutes of Health (Animal Study Protocol #K083-LBC-05). P2Y_6_R-knockout mice were generated by Bar et al. [25], and we used these mice in our studies. Adipocytes were isolated from mouse as described [38]. In short, epididymal fat pads were removed, minced and digested using collagenase for 60–90 min at 37°C in Krebs-Ringer-bicarbonate-HEPES buffer. The stromal vascular fraction was separated from the adipocytes and cultured using DMEM and differentiated as described elsewhere [39]. The differentiated adipocytes were used for the determination of glucose uptake.

### Subcellular fractionation

Membrane fractions were prepared as described with some modifications [40]. Differentiated cells were stimulated with or without P2Y_6_R agonists for 60 min, and then the cells were washed with ice-cold PBS and homogenized in a buffer containing 10 mM Tris/HCl, 1 mM EDTA, 250 mM sucrose (pH 7.4) and protease inhibitors. The homogenate was centrifuged at 3000×g for 2 min. The supernatant was centrifuged at 20,000×g for 20 min and the pellet was suspended in 0.5 ml of the above buffer and layered on top of a liner (15%–35%) sucrose gradient and centrifuged for 60 min at 160,000×g. After the centrifugation, the plasma membrane fraction (1–3.5 cm from the bottom of the tube) was collected and the plasma membrane was pelleted by centrifugation for 60 min at 150,000×g. The pelleted plasma membrane fraction was used for GLUT4 assay by Western blot.

### Western blot analysis

Cell lysates (30 µg protein/well) were analyzed under reducing conditions by SDS-PAGE performed according to Laemmli [41]. Proteins were separated on 12% BisTris gel (Invitrogen, Carlsbad, CA) and transferred to nitrocellulose membranes by electroblotting. Membranes were blocked according to the manufacturer’s instructions and probed with specific antibodies overnight at 4°C. Subsequently, blots were probed with IRdye-conjugated secondary antibody for 1 h and then analyzed using an Odyssey infrared imaging system (Li-COR Biosciences, Lincoln, NE). Unless otherwise mentioned in the figure legends, the blots were scanned and images were captured using the Odyssey imaging system.

### Chromatin immunoprecipitation assay

A ChIP assay was performed on using a kit (Cell Signaling Technology, Danvers,MA) according to the manufacturer’s instructions. Differentiated C2C12 myotubes were treated with P2Y_6_R agonists and/or antagonist, and DNA-protein complexes were cross-linked using 1% formaldehyde for 15 min followed by quenching with 125 mmol/l glycine. Crosslinked chromatins were isolated from the cell lysate using nuclease digestion. HDAC5 was immunoprecipitated using an HDAC5 antibody (Active Motif, Carlsbad, CA) or unrelated antibody (mouse IgG) for controls. DNA was extracted and quantitative PCR was performed with primers designed to amplify GLUT4 promoter containing the MEF2 binding site using the following primers [42]: 5′- CAG GCA TGG TCT CCA CAT ACA C-3′ (forward), 5′-GGT AAC TCC AGC AGG ATG ACA-3′ (reverse).

### Statistical analysis

Results are presented as mean ± SE (n = 3). Assays involving treatment with a single drug were analyzed by 1-way ANOVA with Tukey’s multiple comparison test, and assays using more than one drug were analyzed using two-way ANOVA with Bonferroni post test. Differences between groups were rated significant at a probability error (*P*) <0.05. For the Western blots, the intensity of the bands of the treated samples were calculated based on the intensity of the control sample and then the ratio was calculated to that of the internal controls. The graphical data were analyzed using the nonlinear curve-fitting program Prism 5.0 (GraphPad, San Diego, CA).

## Supporting Information

S1 Fig **Glucose uptake efficacy of P2Y_6_R agonist MRS2957 at different doses in C2C12 myotubes and 3T3-L1 adipocytes.** C2C12 myotubes and 3T3-L1 adipocytes were treated with increasing dosage of MRS2957 and the glucose uptake was evaluated as described in the Materials and Methods section. *P<0.05, when compared to basal.
(PDF)

S2 Fig **Glucose uptake efficacy of MRS2957+Insulin in C2C12 myotubes and 3T3-L1 adipocytes.** C2C12 myotubes and 3T3-L1 adipocytes were co-treated with MRS2957 (100 nM) and insulin at either 200 nM or 500 nM concentration. *P<0.05, when compared to basal; ^@^, not significant when compared to Insulin, 200 nM; ^#^, not significant when compared to insulin, 500 nM; ^&^, *P*<0.05, when compared to MRS2957 (100 nM).
(PDF)

S3 Fig **Glucose uptake with scrambled control siRNA in C2C12 myotubes and 3T3L1 adipocytes.** C2C12 myotubes and 3T3L1 adipocytes were transfected with scrambled control siRNA and was used for the glucose uptake assay after treatment with P2Y_6_R agonist MRS2957 (100 nM) or with MRS2578 (1 µM)+MRS2957 (100 nM). *P<0.05, when compared to controls (n = 3).
(PDF)

## References

[1] Schwartz MW, Seeley RJ, Tschop MH, Woods SC, Morton GJ, et al (2013) Cooperation between brain and islet in glucose homeostasis and diabetes. Nature 503:59–66. 24201279 10.1038/nature12709 PMC3983910

[2] Burnstock G, Verkhratsky A (2012) Purinergic signaling. WIREs Membr Transp Signal 1:116–125 http://dx.doi.org/10.1002/wmts.14

[3] Communi D, Parmentier M, Boeynaems JM (1996) Cloning, functional expression and tissue distribution of the human P2Y_6_ receptor. Biochem Biophys Res Commun 222:303–308. 8670200 10.1006/bbrc.1996.0739

[4] Moore DJ, Chambers JK, Wahlin JP, Tan KB, Moore GB, et al (2001) Expression pattern of human P2Y receptor subtypes: a quantitative reverse transcription-polymerase chain reaction study. Biochim Biophys Acta 1521:107–119. 11690642 10.1016/s0167-4781(01)00291-3

[5] Kim SG, Gao ZG, Soltysiak KA, Chang TS, Brodie C, et al (2003) P2Y_6_ nucleotide receptor activates PKC to protect 1321N1 astrocytoma cells against tumor necrosis factor-induced apoptosis. Cell Mol Neurobiol 23:401–418. 12825835 10.1023/A:1023696806609 PMC3140713

[6] Mamedova LK, Wang R, Besada P, Liang BT, Jacobson KA (2008) Attenuation of apoptosis *in* *vitro* and ischemia/reperfusion injury *in* *vivo* in mouse skeletal muscle by P2Y_6_ receptor activation. Pharmacol Res 58:232–239. 18805489 10.1016/j.phrs.2008.08.004 PMC2586120

[7] Balasubramanian R, Maruoka H, Jayasekara PS, Gao ZG, Jacobson KA (2013) AMP-activated protein kinase as regulator of P2Y_6_ receptor-induced secretion in MIN6 mouse pancreatic β cells. Biochem Pharmacol 85:991–998. 23333427 10.1016/j.bcp.2012.11.029 PMC3594329

[8] Parandeh F, Abaraviciene SM, Amisten S, Erlinge D, Salehi A (2008) Uridine diphosphate (UDP) stimulates insulin secretion by activation of P2Y_6_ receptors. Biochem Biophys Res Commun 370:499–503. 18387359 10.1016/j.bbrc.2008.03.119

[9] Sassmann A, Gier B, Gröne HJ, Drews G, Offermanns S, et al (2010) The Gq/G11-mediated signaling pathway is critical for autocrine potentiation of insulin secretion in mice. J Clin Invest 120:2184–2193. 20440069 10.1172/JCI41541 PMC2877950

[10] Amisten S, Meidute-Abaraviciene S, Tan C, Olde B, Lundquist I, et al (2010) ADP mediates inhibition of insulin secretion by activation of P2Y_13_ receptors in mice. Diabetologia 53:1927–1934. 20526761 10.1007/s00125-010-1807-8

[11] Mamedova L, Joshi BV, Gao ZG, von Kügelgen I, Jacobson KA (2004) Diisothiocyanate derivatives as potent, insurmountable antagonists of P2Y_6_ nucleotide receptors. Biochem Pharmacol 67:1763–1770. 15081875 10.1016/j.bcp.2004.01.011 PMC3413726

[12] Maruoka H, Barrett MO, Ko H, Tosh DK, Melman A, et al (2010) Pyrimidine ribonucleotides with enhanced selectivity as P2Y_6_ receptor agonists: Novel 4-alkyloxyimino, (S)-methanocarba, and 5′-triphosphate γ-ester modifications. J Med Chem 53:4488–4501. 20446735 10.1021/jm100287t PMC2935147

[13] El-Tayeb A, Qi A, Nicholas RA, Müller CE (2011) Structural Modifications of UMP, UDP, and UTP Leading to Subtype-Selective Agonists for P2Y_2_, P2Y_4_, and P2Y_6_ Receptors. J Med Chem 54:2878–2890. 21417463 10.1021/jm1016297

[14] Ginsburg-Shmuel T, Haas M, Schumann M, Reiser G, Kalid O, et al (2010) 5-OMe-UDP is a potent and selective P2Y_6_-receptor agonist. J Med Chem 53:1673–1685. 20095577 10.1021/jm901450d

[15] Merlin J, Evans BA, Csikasz RI, Bengtsson T, Summers RJ, et al (2010) The M_3_-muscarinic acetylcholine receptor stimulates glucose uptake in L6 skeletal muscle cells by a CaMKK-AMPK-dependent mechanism. Cell Signal 22:1104–1113. 20206685 10.1016/j.cellsig.2010.03.004

[16] Song XM, Fiedler M, Galuska D, Ryder JW, Fernström M, et al (2002) 5-Aminoimidazole-4-carboxamide ribonucleoside treatment improves glucose homeostasis in insulin-resistant diabetic (ob/ob) mice. Diabetologia 45:56–65. 11845224 10.1007/s125-002-8245-8

[17] Koistinen HA, Galuska D, Chibalin AV, Yang J, Zierath JR, et al (2003) 5-amino-imidazole carboxamide riboside increases glucose transport and cell-surface GLUT4 content in skeletal muscle from subjects with type 2 diabetes. Diabetes 52:1066–1072. 12716734 10.2337/diabetes.52.5.1066

[18] Kurth-Kraczek EJ, Hirshman MF, Goodyear LJ, Winder WW (1999) 5′ AMP-activated protein kinase activation causes GLUT4 translocation in skeletal muscle. Diabetes 48:1667–1671. 10426389 10.2337/diabetes.48.8.1667

[19] Rachdaoui N, Nagy LE (2003) Endothelin-1-stimulated glucose uptake is desensitized by tumor necrosis factor-alpha in 3T3-L1 adipocytes. Am J Physiol Endocrinol Metab 285:E545–E551. 12773307 10.1152/ajpendo.00160.2003

[20] Karczewska J, Piwkowska A, Rogacka D, Stępiński J, Angielski S, et al (2011) Purinergic modulation of glucose uptake into cultured rat podocytes: effect of diabetic milieu. Biochem Biophys Res Commun 404:723–727. 21163251 10.1016/j.bbrc.2010.12.051

[21] Olson AL, Pessin JE (1996) Structure, function, and regulation of the mammalian facilitative glucose transporter gene family. Annual Rev Nutr 16:235–256. 8839927 10.1146/annurev.nu.16.070196.001315

[22] McGee SL, van Denderen BJ, Howlett KF, Mollica J, Schertzer JD, et al (2008) AMP-activated protein kinase regulates GLUT4 transcription by phosphorylating Histone deacetylase 5. Diabetes 57:860–867. 18184930 10.2337/db07-0843

[23] Vieira RP, Müller T, Grimm M, von Gernler V, Vetter B, et al (2011) Purinergic receptor type 6 contributes to airway inflammation and remodeling in experimental allergic airway inflammation. Am J Respir Crit Care Med 184:215–223. 21512170 10.1164/rccm.201011-1762OC

[24] Warny M, Aboudola S, Robson SC, Sévigny J, Communi D, et al (2001) P2Y_6_ nucleotide receptor mediates monocyte interleukin-8 production in response to UDP or lipopolysaccharide. J Biol Chem 276:26051–26056. 11349132 10.1074/jbc.M102568200

[25] Bar I, Guns PJ, Metallo J, Cammarata D, Wilkin F, et al (2008) Knockout mice reveal a role for P2Y_6_ receptor in macrophages, endothelial cells, and vascular smooth muscle cells. Mol Pharmacol 74:777–784. 18523137 10.1124/mol.108.046904

[26] Guns PJ, Hendrickx J, Van Assche T, Fransen P, Bult H (2010) P2Y receptors and atherosclerosis in apolipoprotein E-deficient mice. Br J Pharmacol 159:326–336. 20050854 10.1111/j.1476-5381.2009.00497.x PMC2825354

[27] Weems JC, Griesel BA, Olson AL (2012) Class II histone deacetylase downregulate GLUT4 transcription in response to increased cAMP signaling in cultured adipocytes and fasting mice. Diabetes 61:1401–1414. 10.2337/db11-0737 PMC3357296 22403301

[28] Sparling DP, Griesel BA, Weems JC, Olson AL (2008) GLUT4 enhancer factor (GEF) interacts with MEF2A and HDAC5 to regulate the GLUT4 promoter in adipocytes. J Biol Chem 283:7429–7437. 18216015 10.1074/jbc.M800481200 PMC2276327

[29] Murgia M, Jensen TE, Cusinato M, Garcia M, Richter EA, et al (2009) Multiple signaling pathways redundantly control glucose transporter GLUT4 gene transcription in skeletal muscle. J Physiol 587:4319–4327. 19596898 10.1113/jphysiol.2009.174888 PMC2754368

[30] Mukwevho E, Kohn TA, Lang D, Nyatia E, Smith J, et al (2008) Caffeine induces hyperacetylation of histones at the MEF2 site on the Glut4 promoter and increases MEF2A binding to the site via a CaMK-dependent mechanism. Am J Physiol Endocrinol Metab 294:E582–E588. 18198354 10.1152/ajpendo.00312.2007

[31] Christensen DP, Dahllöf M, Lundh M, Rasmussen DN, Nielsen MD, et al (2011) Histone deacetylase (HDAC) inhibition as a novel treatment for diabetes mellitus. Med 17:378–390. 10.2119/molmed.2011.00021 PMC3105132 21274504

[32] Inazuka F, Uchida A, Habata Y, Kobayashi M (2006) Compounds controlling P2Y_2_ receptor activity as glycemic agents. PCT Int Appl, WO 2006052007 A1 20060518.

[33] Solini A, Chiozzi P, Morelli A, Passaro A, Fellin R, et al (2003) Defective P2Y purinergic receptor function: A possible novel mechanism for impaired glucose transport. J Cell Physiol 197:435–444. 14566973 10.1002/jcp.10379

[34] Thaning P, Bune LT, Hellsten Y, Pilegaard H, Saltin B, et al (2010) Attenuated purinergic receptor function in patients with type 2 diabetes. Diabetes 59:182–189. 19808895 10.2337/db09-1068 PMC2797920

[35] Lee YJ, Park, Soo Hyun H, Jae H (2005) ATP stimulates Na^+^-glucose cotransporter activity via cAMP and p38 MAPK in renal proximal tubule cells. Am J Physiol 289:C1268–C1276. 10.1152/ajpcell.00002.2005 16014705

[36] Jain S, Ruiz de Azua I, Lu H, White MF, Guettier JM, et al (2013) Chronic activation of a designer G_q_-coupled receptor improves β-cell function. J Clin Invest 123:1750–1762. 23478411 10.1172/JCI66432 PMC3613926

[37] Li JH, Jain S, McMillin SM, Cui Y, Gautam D, et al (2013) A novel experimental strategy to assess the metabolic effects of selective activation of a G_q_-coupled receptor in hepatocytes in vivo. Endocrinology 154:3539–3551. 23861369 10.1210/en.2012-2127 PMC3776870

[38] Ruan H, Zarnowski MJ, Cushman SW, Lodish HF (2003) Standard isolation of primary adipose cells from mouse epididymal fat pads induces inflammatory mediators and down-regulates adipocyte genes. J Biol Chem 278:47585–47593. 12975378 10.1074/jbc.M305257200

[39] Aune UL, Ruiz L, Kajimura S (2013) Isolation and differentiation of stromal vascular cells to beige/brite cells. J Vis Exp Mar 26 (73) doi:10.3791/50191. 10.3791/50191 PMC3641667 23568137

[40] Shibata H, Omata W, Suzuki Y, Tanaka S, Kojima I (1996) A synthetic peptide corresponding to the Rab4 hypervariable carboxyl-terminal domain inhibits insulin action on glucose transport in rat adipocytes. J Biol Chem 271:9704–9709. 8621647 10.1074/jbc.271.16.9704

[41] Laemmli UK (1970) Cleavage of structural proteins during the assembly of the head of bacteriophage T4. Nature 227:680–685. 5432063 10.1038/227680a0

[42] Gong H, Xie J, Zhang N, Yao L, Zhang Y (2011) MEF2A binding to the Glut4 promoter occurs via an AMPKα2-dependent mechanism. Med Sci Sports Exerc 43:1441–1450. 21233771 10.1249/MSS.0b013e31820f6093

