# Supplementary material for: Enhancement of Glucose Uptake in Mouse Skeletal Muscle Cells and Adipocytes by P2Y6 Receptor Agonists
Source: PLoS One. 2014 Dec 30;9(12):e116203. doi: 10.1371/journal.pone.0116203 (PMC4280206; doi:10.1371/journal.pone.0116203)
Supplement: S1 Fig — Glucose uptake efficacy of P2Y6R agonist MRS2957 at different doses in C2C12 myotubes and 3T3-L1 adipocytes. C2C12 myotubes and 3T3-L1 adipocytes were treated with increasing dosage of MRS2957 and the glucose uptake was evaluated as described in the Materials and Methods section. *P<0.05, when compared to basal. (PDF) [file pone.0116203.s001.pdf]

Figure S1. Glucose uptake efficacy of $P2Y_6R$ agonist MRS2957 at different doses in C2C12 myotubes and 3T3-L1 adipocytes

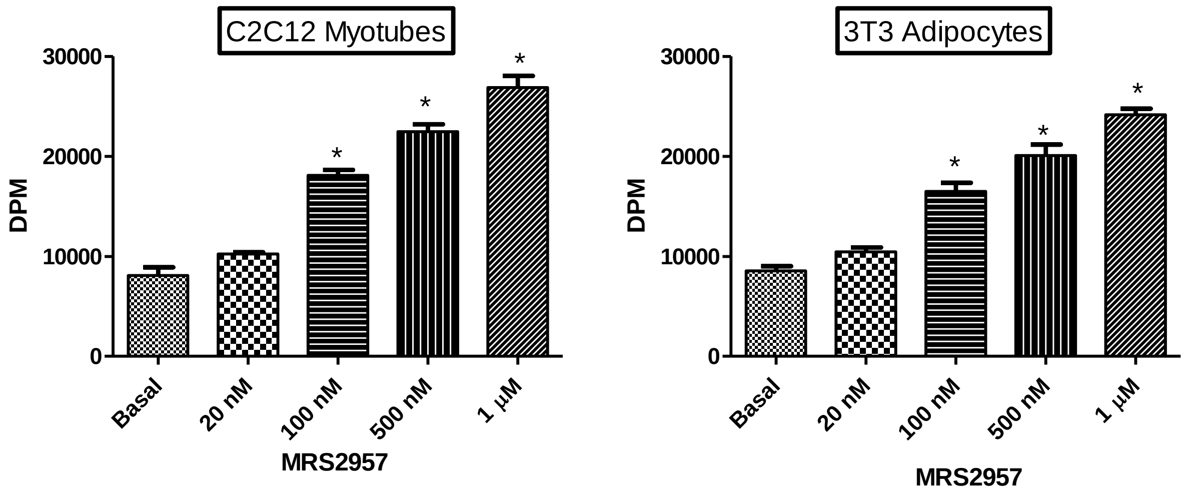

Glucose uptake efficacy of $P2Y_6R$ agonist MRS2957 at different doses in C2C12 myotubes and 3T3-L1 adipocytes. C2C12 myotubes and 3T3-L1 adipocytes were treated with increasing dosage of MRS2957 and the glucose uptake was evaluated as described in the Materials and Methods section.
