# Supplementary material for: Enhancement of Glucose Uptake in Mouse Skeletal Muscle Cells and Adipocytes by P2Y6 Receptor Agonists
Source: PLoS One. 2014 Dec 30;9(12):e116203. doi: 10.1371/journal.pone.0116203 (PMC4280206; doi:10.1371/journal.pone.0116203)
Supplement: S2 Fig — Glucose uptake efficacy of MRS2957+Insulin in C2C12 myotubes and 3T3-L1 adipocytes. C2C12 myotubes and 3T3-L1 adipocytes were co-treated with MRS2957 (100 nM) and insulin at either 200 nM or 500 nM concentration. *P<0.05, when compared to basal; @, not significant when compared to Insulin, 200 nM; #, not significant when compared to insulin, 500 nM; &, P<0.05, when compared to MRS2957 (100 nM). (PDF) [file pone.0116203.s002.pdf]

Figure S2. Glucose uptake efficacy of MRS2957+Insulin in C2C12 myotubes and 3T3-L1 adipocytes

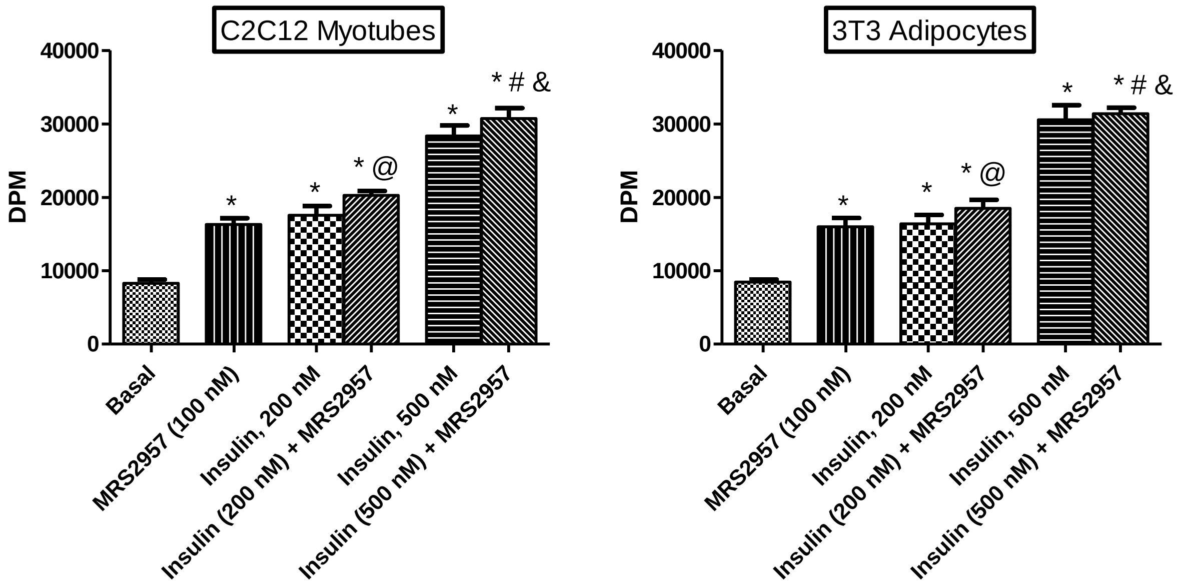

Glucose uptake efficacy of MRS2957+Insulin in C2C12 myotubes and 3T3-L1 adipocytes. *C2C12* myotubes and 3T3-L1 adipocytes were co-treated with MRS2957 (100 nM) and insulin at either 200 nM or 500 nM concentration. *$P<0.05$, when compared to Basal; $^{@}$, not significant when compared to Insulin, 200 nM; $^{\#}$, not significant when compared to Insulin, 500 nM; $^{\&}$, $P<0.05$, when compared to MRS2957 (100 nM).
