# Supplementary material for: Enhancement of Glucose Uptake in Mouse Skeletal Muscle Cells and Adipocytes by P2Y6 Receptor Agonists
Source: PLoS One. 2014 Dec 30;9(12):e116203. doi: 10.1371/journal.pone.0116203 (PMC4280206; doi:10.1371/journal.pone.0116203)
Supplement: S3 Fig — Glucose uptake with scrambled control siRNA in C2C12 myotubes and 3T3L1 adipocytes. C2C12 myotubes and 3T3L1 adipocytes were transfected with scrambled control siRNA and was used for the glucose uptake assay after treatment with P2Y6R agonist MRS2957 (100 nM) or with MRS2578 (1 µM)+MRS2957 (100 nM). *P<0.05, when compared to controls (n = 3). (PDF) [file pone.0116203.s003.pdf]

Figure S3. Glucose uptake with scrambled control siRNA in C2C12 myotubes and 3T3L1 adipocytes

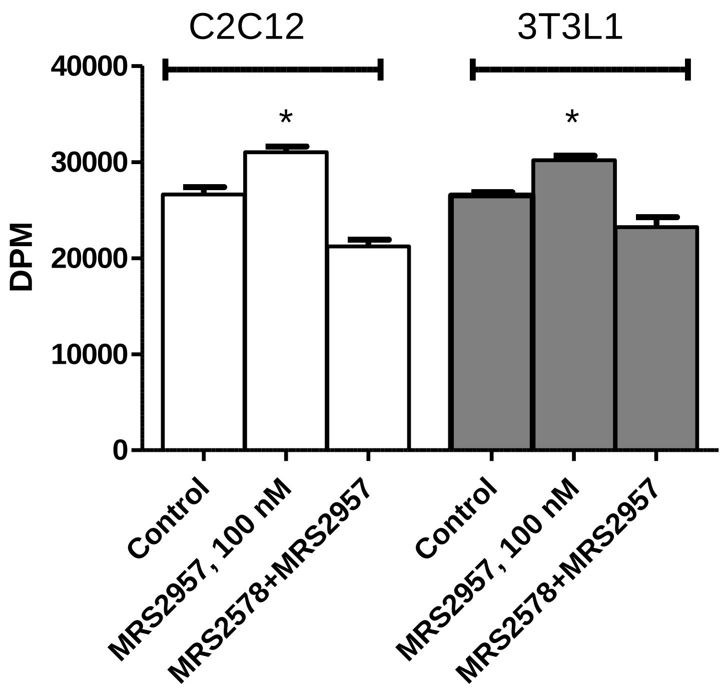

C2C12 myotubes and 3T3L1 adipocytes were transfected with scrambled control siRNA and was used for the glucose uptake assay after treatment with $P2Y_6R$ agonist MRS2957 (100 nM) or with MRS2578 (1 μM)+MRS2957 (100 nM). **P*<0.05, when compared to controls (n=3).
